# NOD2 up-regulates TLR2-mediated IL-23p19 expression via NF-κB subunit c-Rel in Paneth cell-like cells

**DOI:** 10.18632/oncotarget.11467

**Published:** 2016-08-22

**Authors:** Gao Tan, Erbo Liang, Kaili Liao, Feihong Deng, Wendi Zhang, Yuqing Chen, Jun Xu, Fachao Zhi

**Affiliations:** ^1^ Guangdong Provincial Key Laboratory of Gastroenterology, Department of Gastroenterology, Nanfang Hospital, Southern Medical University, Guangzhou, China

**Keywords:** interleukin-23p19, NOD2, Crohn's disease, intestinal immunity

## Abstract

IL-23p19 plays important roles in intestinal antimicrobial immunity, while its over-expression can lead to intestinal inflammation. However, the bacterial compounds and the type of pattern recognition receptor involved in the inducible expression of IL-23p19 in Paneth cells remain unclear. Here we show that the mRNA expression of IL-23p19 was increased in Paneth cell (PC)-like cells stimulated by Toll-like receptor 2 (TLR2) ligands, peptidoglycan (PGN) and Pam3CSK4, and was further increased in the presence of nucleotide-binding oligomerization domain 2 (NOD2)-ligand muramyl dipeptide (MDP). However, its mRNA expression was decreased in NOD2-knockdown PC-like cells. Additionally, the c-Rel activation was increased in Pam3CSK4- or PGN-stimulated PC-like cells, but the PGN-induced c-Rel activation was decreased in NOD2-knockdown PC-like cells and had no significant difference compared with Pam3CSK4-induced c-Rel activation. Our results suggest that NOD2 up-regulates TLR2-mediated IL-23p19 expression via increasing c-Rel activation in PC-like cells. This finding might provide us with a novel therapeutic target for inflammatory bowel disease to inhibit IL-23p19 over-expression via the NOD2-c-Rel pathway.

## INTRODUCTION

Inflammatory bowel disease (IBD) is a chronic and relapsing disorder of the gastrointestinal tract, two clinical phenotypes of which are Crohn's disease (CD) and ulcerative colitis (UC) [1, 2]. Although the exact pathogenesis of IBD remains unknown, available evidence suggests that abnormal T cell responses result in the onset of intestinal inflammation by releasing excessive cytokines that have multiple pathogenic effects on the innate and adaptive immune system [1]. To date, T helper (Th) lymphocytes have been classified into three distinct subsets on the basis of their specific cytokine production profiles, namely Th1, Th2 and Th17 [3, 4]. From this aspect, CD characterized by excessive expression of interferon γ (IFNγ) in inflamed intestine has long been considered to be a Th1 disease [5–9], while UC characterized by abundant IL-17 expression has recently been considered as a Th17 disease [9].

Interleukin (IL)-23, a heterodimeric cytokine, is composed of an IL-23-specific subunit p19 and an IL-12-common subunit p40 [10]. Although IL-12 was predominantly investigated before the discovery of IL-23, many recent studies using IL-23p19^−/−^ mice have identified that IL-23 but not IL-12 has a key role in orchestrating an inflammatory cytokine cascade involving enhanced expressed levels of IL-17, TNFα, IFNγ and IL-6 in the intestine [11–14]. Moreover, a recent study using mucosal specimens from IBD patients has found that the mRNA expression of IL-23p19, but not IL-12p40, was significantly higher in inflamed tissues than in non-inflamed tissues from both CD and UC patients, and the up-regulated mRNA expression of IL-23p19 was significantly correlated with IFNγ in CD and IL17 in UC, suggesting that IL-23p19 may contribute to the intestinal mucosal Th1/Th17 balance in inflammatory bowel disease [9]. Furthermore, some recent studies using blocking antibodies have shown that the spontaneous enteritis in IL-10^−/−^ mice and colonic inflammation in the bacteria-reactive Th17-cell-mediated colitis mice can be significantly ameliorated through treatment with anti-IL-23p19 monoclonal antibodies [15, 16]. However, blockade of IL-23p19 may lead to increased susceptibility to enteric microbial infection, and IL-23p19^−/−^ mice exhibited greatly increased susceptibility and mortality after infection with *Klebsiella pneumonia* [17] and intestinal bacterium *Citrobacter rodentium* [18], suggesting that IL-23p19 serves an important role in mucosal protective immunity. In addition, a recent study using transgenic mice has shown that IL-23p19 over-expression can result in multiple organ inflammation, including intestinal inflammation [19]. Thus, taking control of excessive IL-23p19 expression may be one of the essential factors responsible for novel therapies for IBD and the bacterial compounds and the type of pattern recognition receptor that involved in the inducible expression of IL-23p19 in the intestine deserve fuller exploration.

TLRs are one of the best-characterized pattern recognition receptors (PRRs) that detect conserved microbial components referred to as pathogen-associated molecular patterns (PAMPs) [20, 21]. Up to now, 10 human TLRs have been identified, each of which is composed of N-terminal leucine-rich repeats, C-terminal Toll/IL-1R homology domain and a transmembrane region. Although TLR3 and TLR7-10 are present on endolysosome membrane, TLR1-2 and TLR4-6 are present on plasma membrane. Except for TLR10, the ligands for TLR1-9 have been identified [21–25]. Many studies have shown that TLRs play a major role in the induction of enteric immune responses and can activate multiple pro-inflammatory signaling pathways through the detection of PAMPs to mount an effective bactericidal or antiviral response targeting the invading intestinal microbes [21, 26, 27].

Paneth cells are specialized epithelial cells that function as resident host-defense cells by secreting various mediators [28]. Besides their host defense [29, 30], they could also play a fundamental role in regulating intestinal mucosal immune responses through IL-23p19. Interestingly, these cells constitutively express both IL-23p19 and NOD2 under physiologic conditions and over-express them in CD [31, 32]. Since NOD2 dysfunction is clearly involved in the pathogenesis of CD [33, 34], it would be extremely deserving of investigation whether dysregulated IL-23p19 expression might be due to abnormalities in NOD2 in Paneth cell.

In this study, we used the Paneth cell (PC)-like cells induced as previous methods [35, 36], serving as the functional model of Paneth cells, to investigate the mechanism by which NOD2 may regulate IL-23p19 expression in Paneth cells, since primary Paneth cells do not survive *in vitro* culture [32, 37]. Here we report that NOD2 can up-regulate TLR2-mediated IL-23p19 expression in PC-like cells. In addition, this enhanced effect of NOD2 on IL-23p19 production is caused by increasing nuclear translocation of nuclear factor (NF)-κB subunit c-Rel.

## RESULTS

### TLR2-mediated induction of IL-23p19 expression in PC-like cells

**Figure 1:**
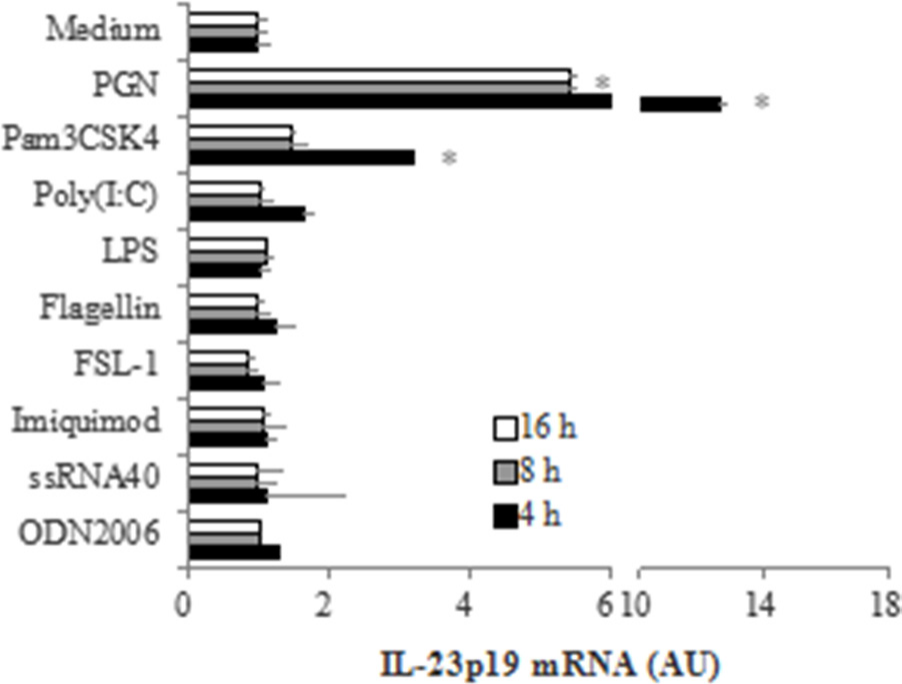
TLR2-mediated induction mRNA expression of IL-23p19 in PC-like cells PC-like cells were stimulated with10 μg/ml PGN, 1 μg/ml Pam3CSK4, 10 μg/ml Poly (I:C), 10 μg/ml LPS, 1 μg/ml Flagellin, 1 μg/ml FSL-1, 1 μg/ml Imiquimod, 1 μg/ml ssRNA40 and 1μM ODN2006 for 4h, 8h and 16h, then total RNA was isolated and IL-23p19 mRNA expression was determined by real-time PCR. Data are normalized to 18 S rRNA and expressed in arbitrary units (AU), representing mRNA induction compared to unstimulated cells. Data are shown as means ± SD of three independent experiments. *P < 0.05 vs. Medium group.

In order to determine which microbial components are capable of inducing IL-23p19 expression in PC-like cells, we stimulated PC-like cells with various bacterial molecules which can interact with host Toll-like receptors (TLRs) (PGN, a TLR2 ligand; Pam3CSK4, a TLR1/2 ligand; LPS, a TLR4 ligand; Flagellin, a TLR5 ligand; FSL-1, a TLR6 ligand; ODN2006, a TLR9 ligand) and some virus-associated TLR-agonists (Poly(I:C), a TLR3 ligand; Imiquimod, a TLR7 ligand; ssRNA40, a TLR8 ligand) and then determined the mRNA expression of IL-23p19 by real-time PCR. We found that the mRNA expression of IL-23p19 was significantly increased in PC-like cells stimulated by PGN and, to a lesser extent, by Pam3CSK4, peaking at 4 h after stimulation (Figure 1). At the peaking time, the mRNA expression of IL-23p19 was ~4-fold higher in PC-like cells stimulated by PGN than by Pam3CSK4 (Figure 1). However, we found that the mRNA expression of IL-23p19 did not significantly increase in PC-like cells stimulated by other non-TLR2 agonists (Figure 1). These results show that activation of TLR2 can induce IL-23p19 expression in PC-like cells. In addition, we also found that the mRNA expression of TNFa and IL-4 was significantly increased in PGN- and Pam3CSK4-stimulated PC-like cells compared with untreated cells (Supplementary Figure S1).

### Up-regulation of TLR2-mediated IL-23p19 expression by NOD2

**Figure 2:**
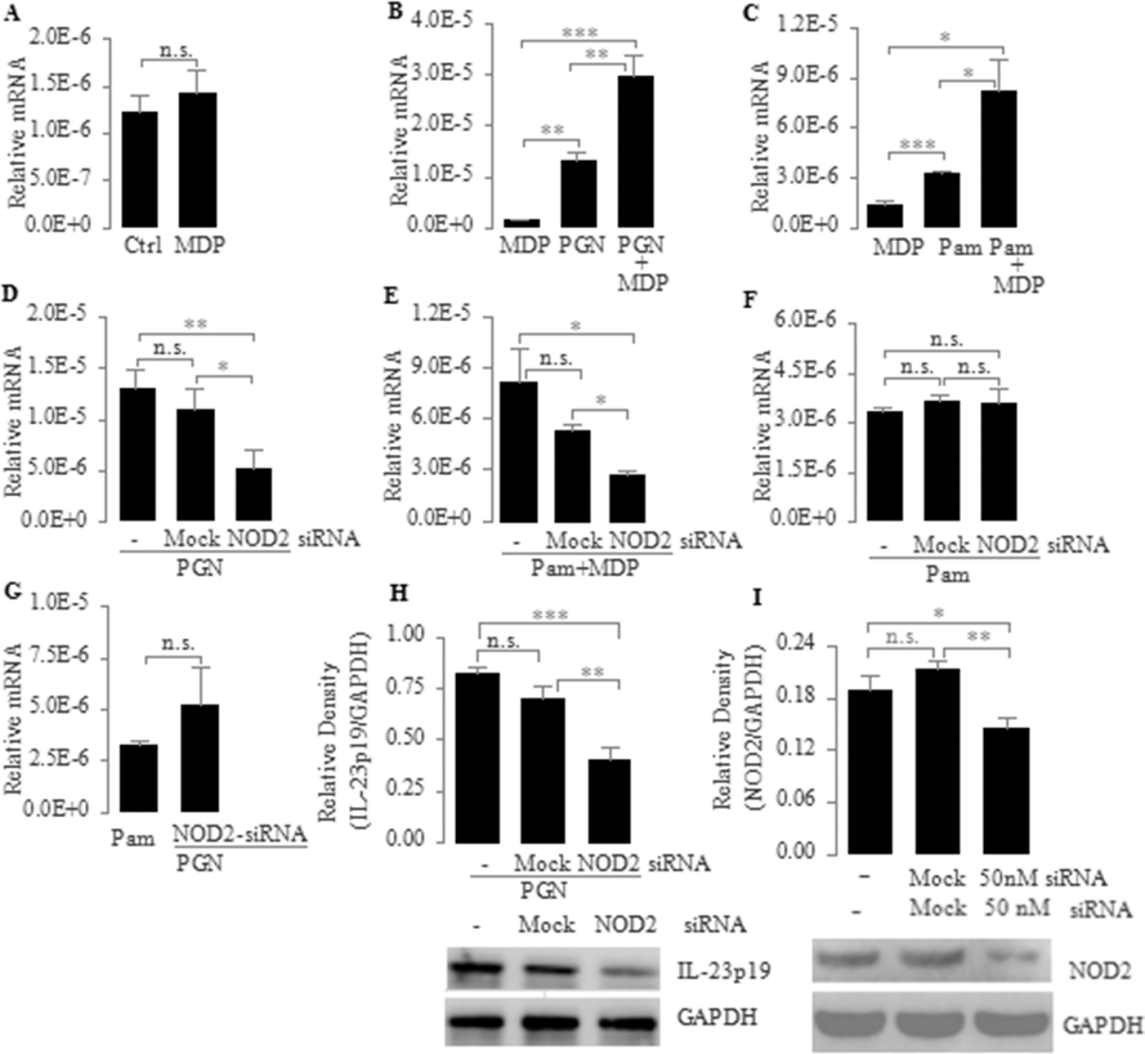
Up-regulation of TLR2-mediated expression of IL-23p19 by NOD2 in PC-like cells **A.** PC-like cells were stimulated for 4 h with MDP (10 μg/ml) only; **B** and **C.** PC-like cells were stimulated for 4 h with the TLR2 agonist PGN (10 μg/ml) (B) and Pam3CSK4 (1 μg/ml) (C) with or without MDP (10 μg/ml); **D-F.** PC-like cells transfected with transfection reagent only (Mock) or NOD2 siRNA (50 nM) were stimulated for 4 h with PGN (10 μg/ml) (D), Pam3CSK4 (1 μg/ml) plus MDP (10 μg/ml) (E) and Pam3CSK4 (1 μg/ml) (F); **G.** PC-like cells were stimulated for 4 h with Pam3CSK4 (1 μg/ml) or PC-like cells transfected with NOD2-siRNA were stimulated for 4 h with PGN (10 μg/ml); (A-G) total RNA was isolated and IL-23p19 mRNA expression was determined by real-time PCR and normalized to 18 S rRNA. **H.** PC-like cells transfected with transfection reagent only (Mock) or NOD2 siRNA (50 nM) were stimulated with PGN (10 μg/ml) for 4 h and finally whole-cell extracts were analyzed for IL-23p19 by western blot. Top, quantitative analysis of proteins; bottom, representative immunoblot images. **I.** Transfection efficiency was tested after 72-h transfection with siRNA by western blot. Ctrl represents untreated PC-like cells. Data are shown as means ± SD of three independent experiments, n.s., not significant; *P < 0.05; **P < 0.01; ***P < 0.001.

Since NOD2 can affect TLR2-mediated responses in various cell types [38, 39], we next addressed the question of whether NOD2 can regulate TLR2-mediated IL-23p19 expression in PC-like cells. To answer this question, we compared the mRNA expression of IL-23p19 in PC-like cells stimulated by TLR2 ligands (PGN, a cell-wall component of bacteria; Pam3CSK4, a pure synthetic agonist) alone and in the presence of MDP. We found that the mRNA expression of IL-23p19 in PC-like cells stimulated by PGN and Pam3CSK4 was significantly higher in the presence of MDP than in the absence of MDP (Figure 2B, 2C). In addition, we also found that IL-23p19 production was greatly enhanced in cultures of PC-like cells stimulated by Pam3CSK4+MDP compared with Pam3CSK4 alone (Supplementary Figure S2). However, the mRNA expression of IL-23p19 did not significantly increase in PC-like cells stimulated by MDP alone (Figure 2A). These results indicate that although activation of NOD2 alone cannot induce IL-23p19 expression, it can up-regulate TLR2-mediated IL-23p19 expression.

To further substantiate the role of NOD2 in regulating TLR2-mediated IL-23p19 expression, we transfected PC-like cells with NOD2-specific small interfering RNA (NOD2-siRNA) and stimulated these transfected cells with PGN, Pam3CSK4 and Pam3CSK4+MDP. We found that the mRNA expression of IL-23p19 induced by PGN and Pam3CSK4+MDP was significantly lower in NOD2-siRNA-transfected PC-like cells than in untransfected PC-like cells or Mock transfectants (Figure 2D, 2E). Consistent with this result, the PGN-induced protein expression of IL-23p19 was significantly lower in NOD2-siRNA-transfected PC-like cells than in untransfected PC-like cells or Mock transfectants (Figure 2H). These results further confirm that NOD2 can up-regulate TLR2-mediated IL-23p19 expression. However, we found that the Pam3CSK4-induced mRNA expression of IL-23p19 did not significantly decrease in NOD2-siRNA-transfected PC-like cells compared with untransfected PC-like cells or Mock transfectants (Figure 2F). This result indicates that Pam3CSK4 does not activate NOD2 in PC-like cells. In addition, we found that there is no significantly difference in the mRNA expression of IL-23p19 between Pam3CSK4-stimulated PC-like cells and PGN-stimulated NOD2-siRNA transfectant (Figure 2G). In conjunction with previous findings that bacterial PGN contains MDP [38, 40, 41], our finding indicates that PGN can activate both cell surface TLR2 and cytoplasmic NOD2 in PC-like cells.

### Induction of TLR2-mediated IL-23p19 expression via the NF-κB pathway

**Figure 3:**
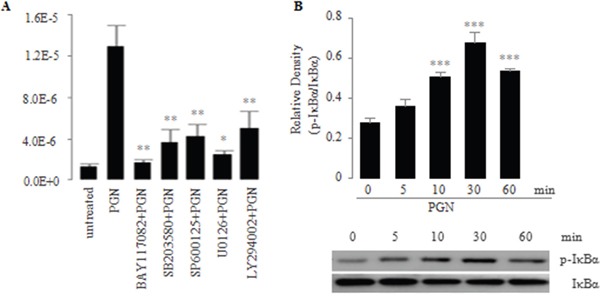
**A.** Effect of signaling inhibitors on PGN-induced expression of IL-23p19 mRNA in PC-like cells PC-like cells were pretreated for 30 min with small molecule inhibitors BAY117082 (10 μM; NF-κB), SB203580 (20 μM; p38 MAPK), SP600125 (50 μM; JNK), and U0126 (50 μM; ERK1/2), LY294002 (50 μM; PI3K) and subsequently were stimulated for 4 h with PGN (10 μg/ml). Total RNA was isolated and IL-23p19 mRNA expression was determined by real-time PCR. **B.** Effect of PGN on activation of IκBα in the NF-κB pathway in PC-like cells. PC-like cells were stimulated with PGN (10 μg/ml), and then whole-cell extracts were prepared at the indicated time points and were analyzed for phospho-IκBα and IκBα by western blot. Top, quantitative analysis of proteins; bottom, representative immunoblot images. Data are shown as means ± SD of three independent experiments. *P < 0.05; **P < 0.01; ***P < 0.001.

Main signal transduction pathways mediating cellular responses to external stimuli are the NF-κB pathway, the PI3K pathway and the MAPK pathway that includes p38, JNK, and ERK1/2 kinases [42]. Therefore, we determined which signaling pathways might be in involved in the TLR2-mediated induction of IL-23p19 expression in PC-like cells. To explore this issue, we pretreated PC-like cells with the specific small molecule inhibitors for NF-κB (BAY11-7082), p38 MAPK (SB203580), JNK (SP600125), ERK1/2 (U0126) and PI3K (LY294002) and then stimulated the pretreated cells with the TLR2 agonist PGN. We found that the PGN-induced mRNA expression of IL-23p19 was most significantly decreased in PC-like cells pretreated by BAY11-7082 (Figure 3A). In addition, we found that IκBα was phosphorylated in response to the concentration of PGN that induced IL-23p19 expression in PC-like cells (Figure 3B). In conjunction with previous findings that IκBα phosphorylation is an essential part of activation of the NF-κB pathway [43], our finding indicates that TLR2-mediated IL-23p19 expression mainly involve the the NF-κB pathway.

### Up-regulation of TLR2-mediated activation of NF-κB subunit c-Rel by NOD2

**Figure 4:**
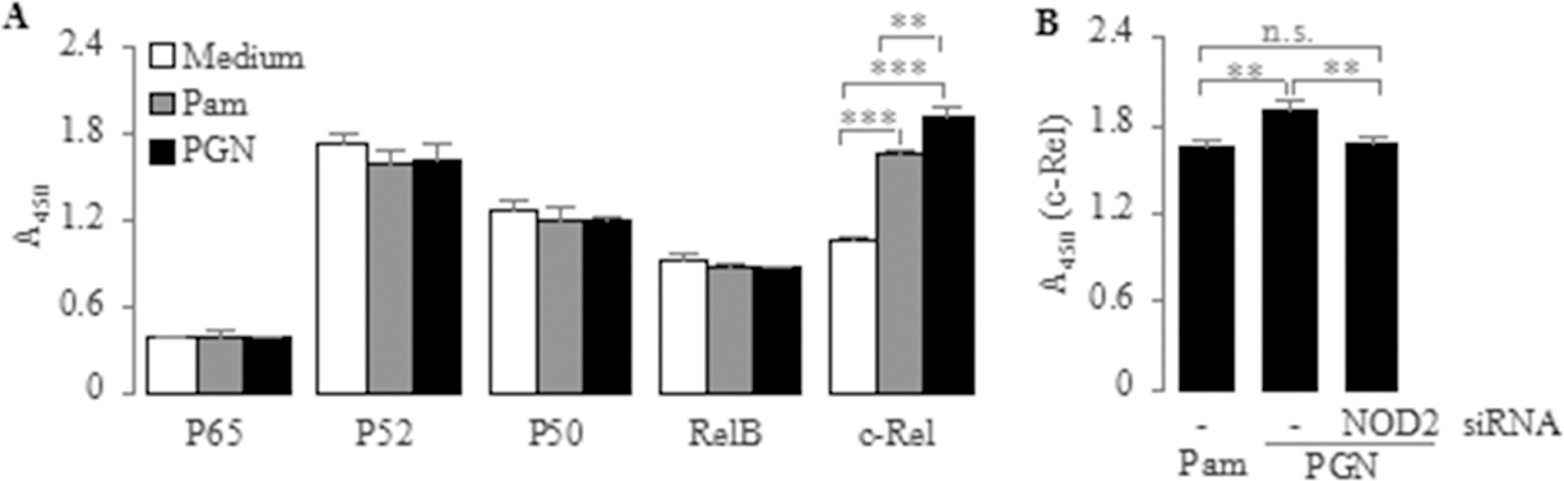
Effect of PGN and Pam3CSK4 on activation of NF-κB subunit c-Rel in PC-like cells **A.** PC-like cells were stimulated for 2 h with Pam3CSK4 (1 μg/ml) and PGN (10 μg/ml), after which nuclear extracts were prepared and translocation of NF-κB subunits (p65, p52, p50, RelB and c-Rel) in nuclear extracts was determined by TransAM assay. **B.** PC-like cells transfected with NOD2-siRNA were stimulated for 2 h with PGN (10 μg/ml), after which nuclear extracts were prepared and translocation of NF-κB subunit c-Rel in nuclear extracts was determined by TransAM assay. Absorbance at 450 nm represents corresponding DNA-binding activity of NF-κB subunits. Data are shown as means ± SD of three independent experiments, n.s., not significant; **P < 0.01; ***P < 0.001.

Since the mammalian NF-κB family is composed of five subunits, namely p65, p52, p50, RelB and c-Rel [43], we next addressed the question of which NF-κB subunits are activated and transduce TLR2 signaling to the nucleus to induce IL-23p19 expression in PC-like cells. To explore this issue, we isolated nuclear proteins from PC-like cells stimulated by Pam3CSK4 and PGN, and then measured the activation of NF-κB subunits in the nuclear extracts. We found that the c-Rel activation was significantly increased in Pam3CSK4- and PGN-stimulated PC-like cells compared with untreated (medium) cells (Figure 4A). However, We found no significant differences in the activation of other NF-κB subunits between Pam3CSK4- and PGN-stimulated PC-like cells and untreated cells (Figure 4A). These results indicate that TLR2 activation induces IL-23p19 expression via NF-κB subunit c-Rel.

Since NOD2 signaling can also result in NF-κB activation [44], we subsequently determined whether NOD2 up-regulates TLR2-mediated IL-23p19 expression via NF-κB subunit c-Rel in PC-like cells. To explore this issue, we compared the c-Rel activation in Pam3CSK4- and PGN-stimulated PC-like cells, as PGN can activate both TLR2 and NOD2 while Pam3CSK4 just is a pure synthetic TLR2 agonist [38]. We found that the c-Rel activation was significantly higher in PGN-stimulated cells than in Pam3CSK4-stimulated cells (Figure 4A). This result suggests that NOD2 activation can up-regulate TLR2-mediated responses probably via increased c-Rel activation in PC-like cells. To further confirm it, we transfected the PC-like cells with NOD2-siRNA and stimulated these transfectants with PGN. We found that the PGN-induced activation of c-Rel was significantly lower in NOD2-siRNA-transfected cells than in untransfected cells (Figure 4B). However, We found no significant differences in the c-Rel activation between PGN-stimulated transfectants and Pam3CSK4-stimulated untransfected cells (Figure 4B). These results prove reversely that NOD2 up-regulates TLR2-mediated IL-23p19 expression via NF-κB subunit c-Rel in PC-like cells.

## DISCUSSION

This study is focused on the mechanism by which NOD2 may regulate IL-23p19 expression in Paneth cells. Our results in PC-like cells show that NOD2 up-regulates TLR2-mediated IL-23p19 expression via NF-κB subunit c-Rel in Paneth cells. In initial studies, we show that although NOD2 by itself cannot induce IL-23p19 expression, it can up-regulate TLR2-mediated IL-23p19 expression. We found that stimulation with the TLR2 agonists PGN and Pam3CSK4 significantly increased the mRNA expression of IL-23p19 in PC-like cells, but stimulation with the NOD2 agonist MDP alone did not. In addition, MDP enhanced PGN- or Pam3CSK4-induced IL-23p19 mRNA expression. However, its mRNA expression was decreased in NOD2-knockdown PC-like cells. Interestingly, We found that compared with Pam3CSK4, PGN stimulation increased IL-23p19 mRNA expression more. However, its mRNA expression was not significant difference between PGN-stimulated NOD2-knockdown cells and Pam3CSK4-stimulated cells. In conjunction with previous findings that bacterial PGN contains MDP [36, 38, 39], our finding provides evidence that PGN can activate both cell surface TLR2 and cytoplasmic NOD2 in PC-like cells.

In further mechanism studies, we show that NOD2 up-regulates TLR2-mediated IL-23p19 expression via increasing activation of NF-κB subunit c-Rel in Paneth cells. We found that although stimulation with PGN and Pam3CSK4 both increased c-Rel activation in PC-like cells, PGN stimulation did more. However, PGN-induced c-Rel activation was decreased in NOD2-knockdown cells. However, the c-Rel activation was not significant difference between PGN-stimulated NOD2-knockdown cells and Pam3CSK4-stimulated cells. These results indicate that NOD2 enhances TLR2-mediated activation of c-Rel.

Some previous studies have suggested that a defective NF-κB pathway due to NOD2 mutations results in a deficient innate immunity, which is responsible for the occurrence of bowel inflammation in CD patients [34, 41, 45, 46]. Nevertheless, this cannot adequately explain the enhanced levels of Th1 cytokines and over-expression of NF-κB molecules in the inflamed tissue of CD patients [47–50]. In contrast, homozygous *NOD2*^2939iC^ mice who harbor the homolog of the most common CD-associated NOD2 variant and even *NOD*^−/−^ mice are healthy and do not cause spontaneous intestinal inflammation [51–53]. Therefore, the loss of NOD2 function becomes difficult to explain the pathogenesis of CD and the gain of NOD2 function in the pathogenesis of CD deserves fuller investigation.

Recent studies show that IL-23p19 has vital roles in the pathogenesis of many chronic inflammatory diseases through activating multiple pro-inflammatory pathways [54, 55], important two of which are the IL-23p19/Th1 pathway in CD and the IL-23p19/Th17 pathway in UC [9]. And some studies show that although appropriate IL-23p19-mediated Th17 responses are protective against bacterial infection [56, 57], excessive Th17 responses serve as precipitating factors of various inflammatory diseases [58]. Here we show that NOD2 can enhance IL-23p19 levels in intestinal tissue, while NOD2 is over-expressed in intestinal tissue from IBD patients [32]. Thus, we suggest that excessive activation of IL-23p19-mediated pro-inflammatory pathways due to NOD2 over-expression may be responsible for the occurrence of intestinal inflammation in IBD patients.

In summary, we show that NOD2 up-regulates TLR2-mediated IL-23p19 expression via NF-κB subunit c-Rel in Paneth-like cells. Some studies show that TLRs play major roles in the induction of enteric immune responses and can activate multiple pro-inflammatory signaling pathways through the detection of PAMPs to mount an effective bactericidal or antiviral response targeting the invading intestinal microbes [21, 26, 27]. These previous findings, in conjunction with our finding, suggest that appropriate IL-23p19 expression induced by activation of TLR2 may meet normal physiological needs to maintain intestinal homeostasis between gut microbes and inflammatory cytokines, while its excessive expression caused by NOD2 may be the major cause for disrupting the intestinal homeostasis and for the occurrence of IBD. Although blocking the activities of IL-23p19 may be the best option in terms of controlling the intestinal inflammatory responses, its huge cost may be damaging their intrinsic beneficial role in host protective immunity [59]. Thus, the NOD2-c-Rel axis of induction of IL-23p19 over-expression may be a better target for development of novel therapies for IBD.

## MATERIALS AND METHODS

### Reagents

Neutralizing monoclonal mouse IgG to human IL-23p19 were purchased from R&D Systems. Neutralizing polyclonal goat antibody to human NOD2 was purchased from Santa Cruz Biotechnology. Monoclonal rabbit antibodies for IκBα and p-IκBα and those for GAPDH were purchased from Cell Signaling Technology. Horseradish peroxidase-conjugated anti-mouse, anti-goat and anti-rabbit IgG secondary antibodies were all purchased from Cell Signaling Technology. TLR ligands (PGN, Pam3CSK4, Poly (I: C), LPS, Flagellin, FSL-1, Imiquimod, ssRNA40 and ODN2006) and NOD2 agonist muramyl dipeptide (MDP) were purchased from InvivoGen. Small molecule inhibitors (BAY117082, LY294002, SB203580, U0126 and SP600125) were purchased from InvivoGen. Recombinant human fibroblast growth factor 9 (FGF9) was purchased from R&D Systems.

### *In vitro* generation and stimulation of Paneth cell (PC)-like cells

Paneth cell (PC)-like cells were generated by treatment of Caco2 cells with FGF9 (10 ng/ml) daily for 3 consecutive days as our described previously [35, 36]. These cells were cultured in Dulbecco's modified Eagle medium (HyClone) supplemented with 10% fetal calf serum (HyClone), 2 mM L-glutamine, 100 U/ml Penicillin, and 100 μg/ml Streptomycin at 37°C in a humidified atmosphere with 5% CO2. For all experiments, to better mimic the steric conditions in the intestine *in vivo*, they were plated at a sub-confluent cell density onto 6-well Millicell hanging filter inserts (3-μm pore size, Polyethylene Terephthalate, Millipore) that allow free access of media to their apical and basolateral sides. Media were changed every 24 h. To test which bacterial compounds can induce IL-23p19 expression in Paneth cells, PC-like cells were stimulated by TLR ligands for 16 h and subsequently harvested and analyzed for IL-23p19 mRNA expression by real-time PCR. To test whether NOD2 can affect IL-23p19 expression, PC-like cells were transfected with NOD2 specific siRNA and then stimulated as indicated in Figure 2. To determine the signaling pathways, PC-like cells were pretreated with small molecule inhibitors for NF-κB (10 μM; BAY117082), PI3K (50 μM; LY294002), p38 (20 μM; SB203580), ERK1/2 (50 μM; U0126), and JNK (50 μM; SP600125) 30 min before treatment with TLR2 ligand PGN (10 μg/ml) and 4 h before IL-23p19 mRNA expression assayed by real-time PCR. For assessment of phospho-IκBα, PGN (10 μg/ml) was added for 5 min, 10min, 30min, and 60min before total protein extraction. PC-like cells (5×10^5^/ml) were stimulated by Pam3CSK4 (1 μg/ml) with or without MDP (10 μg/ml) for 48 h in DMEM containing 10% FCS, then culture supernatants were assayed for IL-23p19 by ELISA kits (ebioscience).

### siRNA

After 24-h culture with antibiotic-free normal growth medium containing 10% fetal calf serum, PC-like cells were transfected with the NOD2 siRNA (50 nM; Santa Cruz Biotechnology) and Transfection Reagent (Santa Cruz Biotechnology) mixture or Transfection Reagent only (Mock) for 6 h, and then incubated with normal growth medium for 18 h. Aspirate the medium, replace with fresh normal growth medium and incubate the cells for an additional 24 h. Subsequently, these cells were stimulated by PGN (10 μg/ml); Pam3CSK4 (1 μg/ml) with or without MDP (10 μg/ml) for 4 h. After that, total RNA was isolated and analyzed by real-time PCR; whole-cell extracts were prepared and analyzed by immunoblotting. Transfection efficiency was tested after 72-h transfection with siRNA by immunoblotting.

### Real-time quantitative RT-PCR analyses

Total cellular RNA was isolated using the RNAiso Plus (Takara Bio) and then cDNA synthesis was performed using the PrimeScript RT reagent Kit with gDNA Eraser (Takara Bio) that can eliminate the genomic DNA contamination. Real-time PCR was performed in triplicate using the LightCycler 480 System (Roche). Each 20-μl PCR reaction contained 5 μl cDNA corresponding to 25 ng RNA as a template, 0.5 μM of each primer, and 1 × LightCycler 480 SYBR Green I Master (Roche). Primer sequences were as follows: IL-23p19: 5′-GTG GGA CAC ATG GAT CTA AGA GAA G-3′, 5′-TTT GCA AGC AGA ACT GAC TGT TG-3′; 18 S rRNA: 5′-TTT GTT GGT TTT CGG AAC TGA-3′, 5′-CGT TTA TGG TCG GAA CTA CGA-3′. Product size: IL-23p19, 124 bp; 18 S rRNA, 199 bp. Samples were loaded into the LightCycler 480 Multiwell Plate 96 (Roche) and incubated for initial denaturation at 95°C for 10 min followed by 45 cycles, each cycle consisting of 95°C for 10 s, “touchdown” of -1°C/cycle from the start annealing temperature 65°C to the end 60°C for 20 s, and 72°C for 20 s. Relative mRNA levels were calculated according the 2^−ΔCT^ method, using 18 S rRNA as the reference and internal standard.

### Immunoblotting

Cells were lysed for 30 min on ice in RIPA lysis buffer (10 mM Tris (pH 8.0), 150 mM NaCl, 1% Nonidet P-40, 0.1% SDS, and 0.5% deoxycholate, supplemented with a protease inhibitor PMSF, and centrifuged at 14,000 g for 30 min at 4°C, and supernatants were collected. SDS-polyacrylamide gel electrophoresis and western blotting were performed in accordance with standard protocols. Antibodies for IL-23p19, IκBα, p-IκBα and GAPDH were all diluted at 1:1000 and antibody for NOD2 at 1:200. Secondary antibodies were all diluted at 1:4000.

### NF-κB activation assay

PC-like cells were treated with MDP (10 μg/ml) for 2 h, after which nuclear extracts were prepared using a Nuclear Extract kit (Active Motif) and the activation of NF-κB subunit p65, p52, p50, RelB and c-Rel in the nuclear extracts was determined using a TransAM NF-κB Family kit (Active Motif). 5 μg nuclear extract was applied to each well coated with NF-κB consensus oligonucleotides, then wells were incubated with rabbit anti-p65, anti-p52, anti-p50, anti-RelB or anti-c-Rel followed by horseradish peroxidase-conjugated anti-rabbit IgG. Finally NF-κB activation was quantified as a colorimetric readout at 450 nm.

### Statistical analysis

Results are shown as means ± standard deviation. Statistical significance was determined by one-way analysis of variance with Tukey's multiple comparisons under equal variances or with Dunnett T3′s multiple comparisons under unequal variances; a value of *P* < 0.05 was considered statistically significant.

## SUPPLEMENTARY MATERIALS FIGURES


