# Supplementary material for: NOD2 up-regulates TLR2-mediated IL-23p19 expression via NF-κB subunit c-Rel in Paneth cell-like cells
Source: Oncotarget. 2016 Aug 22;7(39):63651–60. doi: 10.18632/oncotarget.11467 (PMC5325392; doi:10.18632/oncotarget.11467)
Supplement: Supplementary file 1 [file oncotarget-07-63651-s001.pdf]

NOD2 up-regulates TLR2-mediated IL-23p19 expression via NF-κB subunit c-Rel in Paneth cell-like cells

SUPPLEMENTARY FIGURES

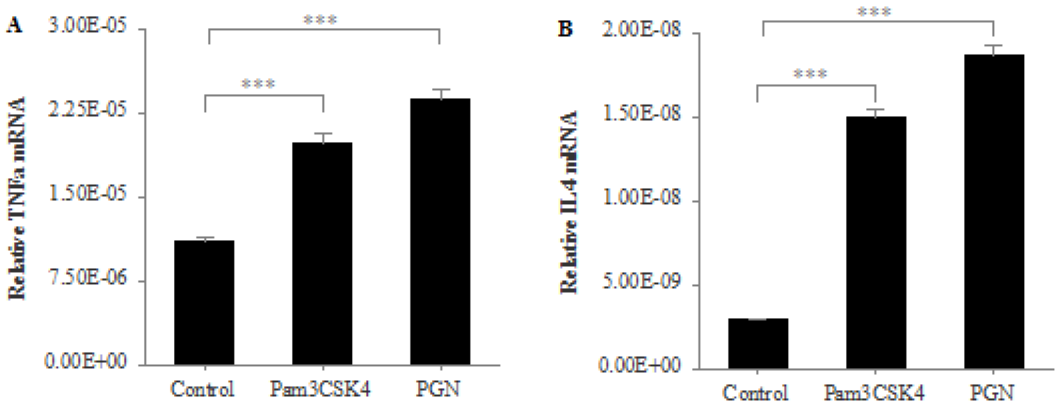

Supplementary Figure S1: TLR2-mediated induction mRNA expression of TNFa A. and IL-4 B. in PC-like cells. PC-like cells were stimulated with10 µg/ml PGN and 1 µg/ml Pam3CSK4 for 4h, then total RNA was isolated and the mRNA expression of TNFa and IL-4 was determined by real-time PCR and normalized to 18 S rRNA. Data are shown as means ± SD of three independent experiments. ***$P \leq 0.001$.

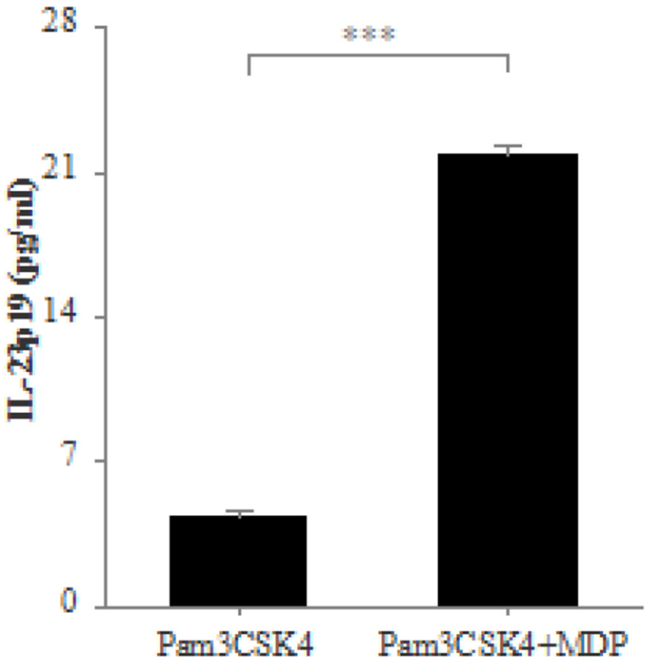

Supplementary Figure S2: Up-regulation of TLR2-mediated expression of IL-23p19 by NOD2 in PC-like cells. PC-like cells (5×10^5/ml) were stimulated by Pam3CSK4 (1 µg/ml) with or without MDP (10 µg/ml). Culture supernatants were collected at 48 h and assayed for IL-23p19 production by ELISA kits (ebioscience). Data are shown as means ± SD of three independent experiments. ***$P \leq 0.001$.
